# Annotations

**Published:** 1899-07-29

**Authors:** 


### Annotations.

### Politics and Profession.

The medical profession is not very largely represented in the House of Commons, and probably most of those medical men who have seats upon its benches are in the habit, on all important occasions, of putting politics before profession. We suppose it must be so. No amount of virtue will secure a man's return to Parliament. Only by politics can he get in, and only by party voting can he keep his place. To ask, then, that our medical representatives in Parliament should combine to form a medical group, as has lately been done by the medical members of the Chamber of Deputies and the Senate in France, and should vote together when medical matters are being considered, would merely be equivalent to asking them to throw up their seats at the next election. Much, however, happens before things come to a vote or even to discussion in the House, and it undoubtedly would be well in all those matters in regard to which the medical members are deferred to as men speaking with authority, such conference should take place between them as should lead them, if possible, not to contradict each other. The differences of doctors are far more puzzling to the laity and more destructive of our influence than anything that we either do or say, and even in their differences our medical M.P.'s are but representative of our much-divided profession. We are told that in France the medical men " drop " party politics when their professional interests are at stake. We see no likelihood of such a consummation in England. Nor is it to be wished. So far politics in this country have to a large extent continued to express the personal opinions of politicians themselves. It is when they can be dropped to serve personal or even professional aims that politics become contemptible.

### The London County Council.

At the meeting of the London County Council held on Tuesday Lord Welby, in reviewing the year's work, touched upon several matters intimately connected with the public health. Whereas between April and December, 1897, there had been 13 cases of mad dogs in the County of London, there had only been one case from January to June, 1898, and from that time to March 31st, 1899, there had not been a single case. The report of the Asylums Committee was one which, he thought, deserved the most earnest consideration, for the annual increase in the number of lunatics under their care amounted to at least 600 each year. One of the most serious points to which he drew attention was the condition of the Thames, which still remained the main source of London's water supply. Although the end of the session had come, the Royal Commission on the water supply had not yet presented their report. Meanwhile, the volume of the water in the river had been falling as compared with previous years, and, indeed, on one day in June it only amounted to 158,000,000 gallons, against 245,000,000, the smallest flow in June, 1898. He thought, and we think, that this is a warning not to be neglected; for whenever it may be decided to bring water from Wales the execution of the work will occupy nine or ten years, and, as he said, time is passing. Another subject on which he touched was the housing of the working classes, a matter of the most vital importance, not alone to the individuals more especially dealt with in any particular scheme, but to the health of the whole metropolis. We ourselves can have but little doubt that crowding the working classes into vast blocks of dwellings must have an injurious influence upon the general public health, unless the process be combined with such a degree of disciplinary control as will interfere greatly with the freedom and the responsibility of home life on which so much of our national character depends, and we feel that Lord Welby is on the right tack when he looks hopefully to the building of dwellings in districts easily accessible by rail or tram, rather than crowding up the central parts of London. So far as health is concerned, London wants to spread rather than to concentrate; and so far as *morale*, to become a county of homes rather than a city of lodgings.

### The Metropolitan Asylums Board and its Work.

The annual report of the Metropolitan Asylums Board is a document which is of far more than mere medical interest. The vast work being done by the Board in the removal, the isolation, and the treatment of persons suffering from infectious disease is so constantly and prominently brought before the public, and from the medical standpoint so far overshadows all the rest, that we are, perhaps, too apt to forget how many other departments of usefulness are served by the Board. What with imbeciles, fever and small-pox patients, convalescents, boys, and convalescent and defective children, it has a grand total of 13,537 persons, who, if all the accommodation were occupied, would have to be provided for day by day; some, as in the imbecile asylums, to be merely nursed through their miserable and hopeless lives; some, as in the schools attached to these asylums, to have their feeble minds educated as so far as education may be possible for them; some, as in the great hospitals, to be carefully treated while suffering from acute infectious diseases, and to be so isolated as to prevent the spread of infection. Some, again, as the lads on board the " Exmouth," to be trained up to lead useful lives instead of being cast upon the streets; while others, as at St. Anne's Home and East Cliff House, to be restored to more complete health than would be possible in the smoke and grime of a great city; and still others, again, as at Lloyd House, Pentonville, to be placed within reach of such educational advantages as the Board's school system is able to offer to defective children. But even that does not cover the whole work of the Board, which is now responsible for the care of about 800 children suffering from ophthalmia and 400 from ringworm and other contagious diseases of the skin, and also for an average of about 150 children who hitherto have been sent to workhouses under remand by the magistrates. The Metropolitan Asylums Board has been the willing horse on to whose back all sorts of difficult problems have been cast. By dint of energy, and efficient officers, it has served the public well, nor does it seem unwilling to add even still further to its burdens. Whether, however, a body so constituted is quite the proper body to undertake the very varied work which it is at present doing, is another question.

